# Team effectiveness: epidemiologists’ perception of collective performance during emergency response

**DOI:** 10.1186/s12913-023-09126-y

**Published:** 2023-02-13

**Authors:** Amy Elizabeth Parry, Alice Richardson, Martyn D. Kirk, Samantha M. Colquhoun, David N. Durrheim, Tambri Housen

**Affiliations:** 1 The Australian National University (ANU), National Centre for Epidemiology & Population Health (NCEPH), Building 62 Mills Road, Acton, ACT Australia; 2 The Australian National University, Statistical Support Network, Acton, Australia; 3 University of Newcastle, Newcastle, NSW Australia

**Keywords:** Epidemiology, Public health practice, Health workforce, Emergency, Leadership, Teamwork

## Abstract

**Background:**

To describe epidemiologists’ experience of team dynamics and leadership during emergency response, and explore the utility of the Team Emergency Assessment Measure (TEAM) tool during future public health emergency responses. The TEAM tool included categories for leadership, teamwork, and task management.

**Methods:**

We conducted a cross-sectional survey between October 2019 and February 2020 with the global applied field epidemiology workforce. To validate the TEAM tool for our context, we used exploratory and confirmatory factor analysis.

**Results:**

We analysed 166 completed surveys. Respondents included national and international emergency responders with representation of all WHO regions. We were unable to validate the TEAM tool for use with epidemiology teams involved in emergency response, however descriptive analysis provided insight into epidemiology emergency response team performance. We found female responders were less satisfied with response leadership than male counterparts, and national responders were more satisfied across all survey categories compared to international responders.

**Conclusion:**

Functional teams are a core attribute of effective public health emergency response. Our findings have shown a need for a greater focus on team performance. We recommend development of a fit-for-purpose performance management tool for teams responding to public health emergencies. The importance of building and supporting the development of the national workforce is another important finding of this study.

## Background

The impact of the COVID-19 pandemic has accentuated the importance of an effective emergency response epidemiology workforce. Public health emergencies, like COVID-19, are expected to increase in frequency and severity due to factors such as climate change and urbanisation [1]. We must heed this evidence to ensure an optimal response.

The epidemiology emergency response workforce aims to reduce the impact of a crisis on affected populations [2]. The effectiveness of response teams is critical to mitigating this impact. In this study, we consider a team as a group of people who work together to achieve a set outcome, and an effective team as one who collaborates, produces a desired result, and are collectively competent [3, 4]. This study focused on the experience of the epidemiological workforce within multi-disciplinary emergency response teams [5].

Team dynamics underpin team effectiveness. Tuckman’s seminal work on teams identified five stages of a team lifecycle [6]. The initial stage, ‘forming’, is where initial relationships are developed. ‘Storming’ is reported to be a more turbulent stage, where the team challenge each other and can conflict on issues and in team relationships. ‘Norming’ is where the team develop standards, roles, and feel more comfortable sharing opinions. ‘Performing’ is when the team relationships are stable, roles are clear, and the team are able to focus on tasks and solve problems together. Over time, and through these stages, the team learn become more effective until the end of the team lifecycle, ‘Adjourning’ [6].

Team effectiveness is also influenced by team competence and team management. Team competence is established through delineation of clear roles, clarification of expectations, and development of a shared understanding of skills, knowledge, and motivation [7]. Team management in response is hampered by short deployment periods, high staff rotation, limited standardisation of epidemiology training or skills, and challenges associated with leadership and communication [4, 8–11]. The combined impact of team dynamics, team confidence and team management during emergency response influence the teams effectiveness [12].

Valid and reliable teamwork assessment tools may highlight factors requiring intervention to strengthen individual and team performance. Numerous tools are available to assess teams during a crisis, however, they have largely been developed for clinical settings and are not routinely used during public health emergency response events [13]. A systematic review of teamwork measurement tools during crises compared 13 tools [13]. One of those 13 tools was the Team Emergency Assessment Measure (TEAM). The TEAM was originally developed for use in hospitals to evaluate team performance during resuscitation events, and was found to provide valid and reliable team performance assessment with respect to leadership, teamwork, and task management during emergency response in the hospital environment (Table 1) [4]. The generalisability of this tool to alternate emergency response environments has not been assessed [13–18]. We selected the TEAM tool for our survey as the ‘elements’ (Table 1) reflected issues raised by our key informant interviewees,[4] and to test the generalisability of the tool in an epidemiology emergency response setting.

**Table 1:** The Team Emergency Assessment Measure (TEAM) categories and elements [19, 20].

Categories	Elements	Items
Leadership	Leadership	1.The team leader let the team know what was expected of them through direction and command
	Control	2. The team leader maintained a global perspective
Teamwork	Communication	3. The team communicated effectively
	Co-operation and Co-ordination	4. The team worked together to complete the tasks in a timely manner
	Team climate	5. The team acted with composure and control
	Team climate	6. The team morale was positive
	Adaptability	7. The team adapted to changing situations
	Situation awareness/ perception	8. The team monitored and reassessed the situation
	Situation awareness/ projection	9. The team anticipated potential actions
Task management	Prioritisation	10. The team prioritised tasks
	Clinical standards	11.The team followed approved standards and guidelines
Global rating		12. On a scale of 1–10 give your global rating of the team’s non-technical performance

## Methods

### Study design

We developed and distributed a cross sectional survey to investigate epidemiologists’ experience of team dynamics and leadership during emergency responses. This survey was part of a larger study, conducted prior to the COVID-19 pandemic [4, 5, 8].

### Study population

The global applied epidemiology workforce was the target population for this survey. We defined this workforce as any person working in an applied or field epidemiology role or acute public health responder role. For this analysis, we analysed responses of participants who indicated experience in responding to emergencies in an epidemiology role.

### Recruitment

We applied purposive and snowballing sampling techniques to identify potential participants [21, 22]. We collaborated with TEPHIConnect, an online platform of international Field Epidemiology Training Program (FETP) alumni, to distribute our survey to 1700 registered members (September 2019). We ran a social media campaign and advertised the survey at international epidemiology conferences in both English and French over a three-month period, October 2019 – February 2020.

### Data collection

The survey was self-administered online via REDCap (Research Electronic Data Capture). We selected the Team Emergency Assessment Measure (TEAM) tool (Table 1) [19] as it aligned with needs identified during key informant interviewees [4]. Questions 1–11 used a Likert scale format with a five-point scale ranging from 0 (never/hardly ever) to 4 (always/nearly always). These questions covered three overarching categories; leadership, teamwork, and task management. The final TEAM question used a scale from 0 to 10 for participants to rate the team’s overall performance [20]. We did not change the wording of the questions, however we made minor wording adjustments to the question prompts to ensure appropriateness to the epidemiologist context. We added one additional open ended question to obtain individual perspectives on team functionality during emergency response. Participants were asked to answer the questions reflecting on their most recent emergency response prior to survey completion.

### Data analysis

To calculate the overall score, we used the TEAM tool rating scale [19, 20]. According to the TEAM tool, a total performance score on the first 11 questions of 33 or less was considered “poor”, 34–39 was “good” and 40–44 was “excellent” [19]. In the final question, participants were asked to provide an overall ‘global’ rating of team performance. An overall rating of 8–10 was considered “excellent”, 7 or less was “poor” [19].

We defined those who reported their most recent emergency response within a national setting as ‘national responders’, and those who reported their most recent emergency response within an international setting as ‘international responders’.

Survey data was analysed using Microsoft Excel (2016) and Stata15 (TX:StataCorp). To explore differences between responder types (international or national) and self-identified gender we conducted either the Student t-test or Kruskal–Wallis test depending on the nature of the data, results are shown as t-test. We considered differences significant if they fell outside the 95% confidence interval. Content analysis was used to analyse responses to open-ended questions.

To investigate the usefulness of the TEAM tool for this context, we first conducted an exploratory factor analysis. We measured internal consistency of items using Cronbach’s alpha, where a value of > 0.7 was considered consistent [23–26]. We then conducted a confirmatory factor analysis assuming three factors. Factor loadings are the correlation between an item and a factor, we considered factor loadings > 0.5 to be interpretable [24].

### Consent and ethics

This survey was approved by The Australian National University Human Research Ethics Committee, ID 2019–068.

## Results

### Demographics

We analysed 166 completed surveys. The median age of respondents was 39 years (range: 23–77 years), and 51% (*n* = 85/166) identified as female. All World Health Organization (WHO) regions were represented (Table 2).

**Table 2:** Demographic characteristics of epidemiology emergency response survey respondents, 2019–2020

Category	Items	*n* = 166 (100%)
Survey language	English	157 (95%)
	French	9 (5%)
Age (years)	< 20	0 (0%)
	20–29	10 (6%)
	30–39	78 (47%)
	40–49	47 (28%)
	50–59	25 (15%)
	60 +	6 (4%)
Identified gender	Female	85 (51%)
	Male	79 (48%)
	Not reported	2 (1%)
Region (WHO region)	Africa	43 (26%)
	Americas	47 (28%)
	Eastern Mediterranean	6 (4%)
	Europe	20 (12%)
	South-east Asia	14 (9%)
	Western Pacific	36 (22%)
Responder type (*n* = 162/166)	National	96 (59%)
	International	66 (41%)
Epidemiology experience (n = 164/166)	< 5 years	53 (32%)
	5 + years	113 (68%)
Epidemiology emergency response experience (*n* = 164/166)	≤ 3 events	79 (48%)
	4 + events	85 (52%)

^*^denominator = *166 unless otherwise stated*

The survey asked about the respondents’ experience during their most recent emergency response; 96 (59%) reported they had responded within their own country, 66 (41%) had been deployed internationally.

### The team emergency assessment measure (TEAM)

The mean overall score in this survey was 32 of a possible 44 (range 0–44), with a standard deviation of 7.4. The mean score for national responders was 34 (range 8–44), and international responders was 29.5 (range 0–44) (*p* = 0.001) (Table 2).

Comparing the three tool categories, ‘task management’ scored the highest overall rating and ‘leadership’ scored the lowest, however, there was little variation between the final assessment scores (Table 3).

**Table 3:** Team Emergency Assessment Measure (TEAM) rating, epidemiology emergency response survey, 2019–2020 (*n* = 166)

			**Gender**				**Responder type**					
**No**	**Item**	Total mean/SD** (*n* = -166)	Female Mean/SD** (*n* = 85)	Male Mean/SD** (*n* = 79)	**t**	***p*-value**	Nat^ rating range*	Nat^ mean/ SD** (*n* = 96)	Int^ rating range* (*n* = 66)	Int^ mean/ SD**	**t**	***p*-value**
**Leadership average (Q1-Q2)**		**2.8** ± **0.9**	**2.6 ± 1.1**	**2.9 ± 0.8**	**-2.2**	**0.03**	**0–4**	**3.0 ± 0.8**	**0–4**	**2.5 ± 1.1**	**-2.9**	**0.003**
Q1	The team leader let the team know what was expected of them through direction and command (*n* = 152)	3.0 ± 1.0	2.7 ± 1.2	3.2 ± 0.8	-2.9	0.005	1–4	3.2 ± 0.8	0–4	2.6 ± 1.1	-3.3	0.001
Q2	The team leader maintained a global perspective (*n* = 150)	2.6 ± 1.1	2.5 ± 1.2	2.7 ± 1.0	-1.3	0.18	0–4	2.8 ± 1.1	0–4	2.4 ± 1.1	-1.9	0.05
**Teamwork average (Q3-Q9)**		**2.9 ± 0.7**	**2.8 ± 0.8**	**2.9 ± 0.6**	**-1.1**	**0.27**	**0–4**	**3.0 ± 0.6**	**0–4**	**2.7 ± 0.8**	**-2.9**	**0.004**
Q3	The team communicated effectively (*n* = 150)	2.9 ± 0.9	2.7 ± 1.0	3.1 ± 0.8	-2.5	0.01	0–4	3.1 ± 0.8	0–4	2.6 ± 1.0	-3.8	0.0002
Q4	The team worked together to complete the tasks in a timely manner (*n* = 144)	3.1 ± 0.9	2.9 ± 0.9	3.2 ± 0.7	-2.0	0.05	0–4	3.3 ± 0.7	0–4	2.8 ± 1.0	-3.7	0.0002
Q5	The team acted with composure and control (*n* = 149)	3.0 ± 0.8	2.9 ± 0.9	3.0 ± 0.7	-0.2	0.84	1–4	3.1 ± 0.8	0–4	2.8 ± 0.9	-2.2	0.03
Q6	The team morale was positive (*n* = 149)	2.8 ± 0.9	2.8 ± 0.9	2.9 ± 0.8	-0.5	0.59	1–4	3.0 ± 0.8	0–4	2.6 ± 0.9	-2.5	0.01
Q7	The team adapted to changing situations (*n* = 149)	2.9 ± 0.8	2.9 ± 0.9	2.9 ± 0.9	0.1	0.91	1–4	3.0 ± 0.8	0–4	2.8 ± 0.8	-2.0	0.05
Q8	The team monitored and reassessed the situation (*n* = 148)	2.9 ± 0.9	2.9 ± 0.9	3.0 ± 0.9	-0.4	0.65	0–4	3.0 ± 0.9	0–4	2.9 ± 0.9	-0.8	0.41
Q9	The team anticipated potential actions (*n* = 147)	2.8 ± 0.9	2.7 ± 0.9	2.8 ± 0.9	-0.8	0.41	0–4	2.9 ± 0.9	0–4	2.6 ± 0.9	-1.9	0.05
**Task management average (Q10-Q11)**		**3.1** ± **0.8**	**3.0 ± 0.8**	**3.2 ± 0.8**	**-1.5**	**0.13**	**1–4**	**3.2 ± 0.7**	**0–4**	**2.8 ± 0.9**	**-3.0**	**0.003**
Q10	The team prioritised tasks (*n* = 149)	3.1 ± 0.8	3.0 ± 0.9	3.1 ± 0.8	-1.1	0.29	1–4	3.2 ± 0.7	0–4	2.8 ± 0.9	-3.1	0.002
Q11	The team followed approved standards and guidelines (*n* = 148)	3.0 ± 0.8	2.9 ± 0.9	3.2 ± 0.7	-1.7	0.08	1–4	3.2 ± 0.7	0–4	2.8 ± 1.0	-2.4	0.02
**Total TEAM score (Q1-11)**		**32 ± 7.4**	**31 ± 8.3**	**33 ± 6.4**	**-1.5**	**0.14**	**12–44**	**34 ± 6.1**	**0–44**	**29.5 ± 8.5**	**-3.3**	**0.001**

^*^Rating = 0 = never/hardly ever, 1 = seldom, 2 = about as often as not, 3 = very often, 4 = always/nearly always

^**^SD = standard deviation

^Nat = National, Int = International

National responders reported statistically higher satisfaction in all survey questions compared to international responders, with the exception of question eight: *‘monitoring and assessing the situation’* (Table 3). No statistical difference was found between epidemiology experience, epidemiology training type, or years of epidemiology experience (data not shown).

### Leadership

Participants were asked about the leadership direction and perspective during their last response. The average leadership score across both questions and all respondents was 2.8 of a possible 4. For both leadership questions, respondents of national teams reported statistically significant higher satisfaction on average than those of international teams (*p* = 0.003). Male respondents rated leadership more highly than females (*p* = 0.03) (Table 3).

The first question on leadership direction addressed whether team leaders were clear about their direction and command, and whether the team knew what was expected. National responders reported higher satisfaction in leadership direction (*p* = 0.001). Similarly, male respondents were more satisfied with leadership direction and command (*p* = 0.005) (Table 3).

The second leadership question asked whether the team leader maintained a ‘global perspective’. National responders reported higher satisfaction with leadership perspective than international responders (*p* = 0.05) (Table 3).

In free text responses, respondents reported needing more leadership training. Respondents indicated that people with strong technical skills were often placed in positions of leadership, but had limited skills in this role. *“It is critical for the team leader to have operational and logistics training and experience. It's not always about the science, but surviving the environment around the science.”*

Gaps in leadership during response were frequently reported where team members needed to unofficially step up into the role. *“We did not have a lead epi[demiologist] the entire time I was there. So the epi[demiology] team just managed ourselves together”* and *“we had a very poor leader, however, the team was very strong and had worked through similar outbreak responses together so there were pseudo-leaders who were able to step up and lead the team.”*

### Teamwork

Participants were asked seven questions related to teamwork; communication, cooperation, coordination, team climate, adaptability, situation awareness regarding perception and projection. The teamwork mean rating was 2.9/4, and national responders reported a higher rating than international responders (*p* = 0.004).

For each of the seven questions national responders reported higher satisfaction than international responders. With the exception of question eight (monitoring and assessing situation), there was a statistically significant difference of teamwork ratings between national and international responders for each question (*p* =  < 0.05). *“Team membership changes a lot, lots of different people cycling through with limited direction provided. It was difficult for the external team and home country team to have so much change and lack of consistency.”*

The highest rating within teamwork was in working together to complete tasks in a timely manner. Males reported high satisfaction relating to communication and collaboration (*p* =  < 0.05). Effective communication was one of the most discussed teamwork topics. *“Our group was good, but communication and direction from those above us was not well coordinated. Roles and expectations often not clear,”* and *“Communication and collaboration is the key”.*

The lowest scores within the teamwork category were regarding morale and anticipating actions. One responder stated *“some team members were not committed and some lacked confidence and capabilities so needed to constantly be supervised”* and another commented on the need for laughter *“surviving and laughing about the austere conditions and very long hours.”*

Timely completion of work was raised as an issue, with one responder stating *“I feel it was more kind of damage control exercise, if we have acted more swiftly we could have done better.”*

### Task management

Task management was considered in two categories; prioritisation of tasks and following standards and guidelines. National responders once again reported significantly higher satisfaction than international responders (*p* = 0.003).

Respondents highlighted the cyclical nature of emergency response teams as a challenge for both teamwork as well as task management, *“[I] saw things being repeated unnecessarily.”*

### Overall ‘non-technical’ rating

**Fig. 1:**
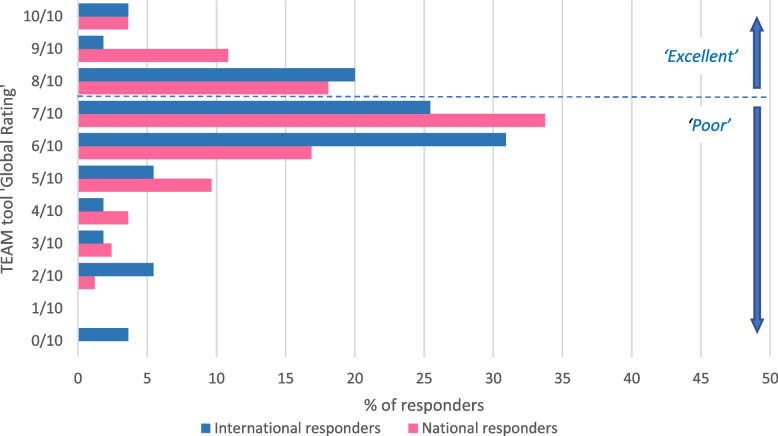
Rating of overall team non-technical performance, Team Emergency Assessment Measure (TEAM) tool epidemiology emergency response survey 2019–2020 (*n* = 166)

The final question provided a scale from 0 to 10 for participants to rate the overall performance of their epidemiology team [19]. Of those who answered (n = 138/166), the mean and median scores were both 7/10 (range 0–10). The TEAM tool states a score of 7 or less is considered ‘poor’; 70% (97/138) of participants in this survey gave an overall rating of 7 or less (Fig. 1). There was no statistical difference in scores between national and international responders for this question.

### TEAM tool verification and reliability

**Fig. 2:**
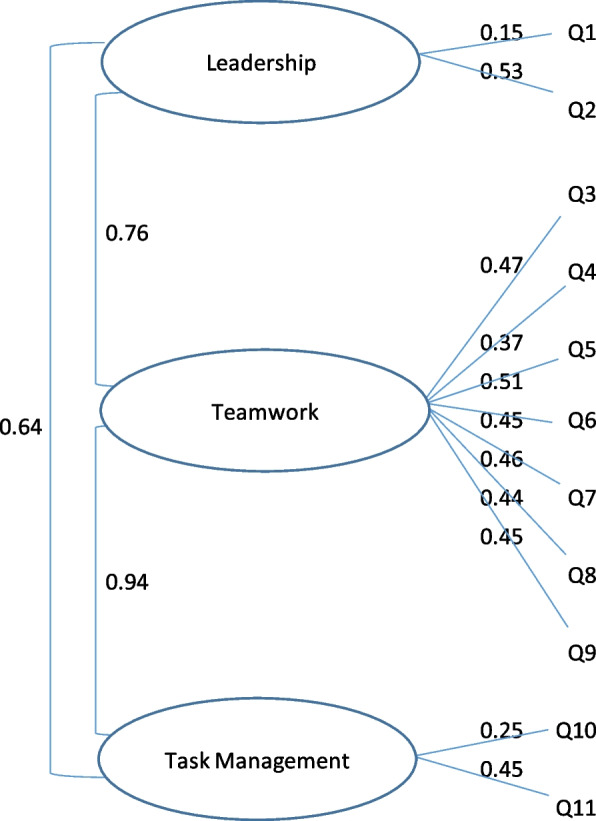
Confirmatory factor analysis findings, Team Emergency Assessment Measure (TEAM) tool use in epidemiology emergency response team performance

The TEAM tool comprised three latent variables (inferred rather than directly observed variables); leadership, teamwork, and task management. Our exploratory factor analysis identified only two latent themes, with the original teamwork and task management being highly correlated (Table 4). The Cronbach alpha for the two identified factors indicated acceptable internal consistency for two factors (Table 4). Our confirmatory factor analysis found that the factor loading on most questions was under 0.5 (Fig. 2), therefore not reflecting the three latent variables identified in the original tool [19]. Although face validity appears to be supported by the two factor solution, the confirmatory factor analysis findings indicate that the questionnaire does not measure its intended concepts in our survey and study population.

**Table 4:** Factor structure of Team Emergency Assessment Measure (TEAM) tool, by exploratory factor analysis

Item	Factor 1	Factor 2
Q9. The team anticipated potential actions	0.82	0.16
Q10. The team prioritised tasks	0.81	0.28
Q8. The team monitored and reassessed the situation	0.78	0.24
Q11.The team followed approved standards and guidelines	0.77	0.17
Q6. The team morale was positive	0.65	0.42
Q7. The team adapted to changing situations	0.62	0.45
Q4. The team worked together to complete the tasks in a timely manner	0.61	0.55
Q5. The team acted with composure and control	0.55	0.49
Q2. The team leader maintained a global perspective	0.07	0.87
Q1.The team leader let the team know what was expected of them through direction and command	0.33	0.80
Q3. The team communicated effectively	0.499	0.61
**Cronbach alpha by factor**	**0.92**	**0.79**

## Discussion

We described epidemiologists’ experiences of team dynamics and leadership, and tested the utility of the TEAM tool for use during public health emergency response. Unfortunately, the tool tested in this study did not satisfactorily perform in our emergency response setting, we were unable to verify the three themes of leadership, teamwork, and task management through either confirmatory or exploratory factor analysis. Although the face validity of the TEAM tool appears to suitably address the identified latent variables, the confirmatory factor analysis findings indicate that the questionnaire did not measure its intended concepts in our survey and study population. Despite being unable to validate the TEAM tool in this setting, we gained important insights into emergency response team performance from the perspective of epidemiologists.

### Performance management

The effectiveness of emergency response teams is critical to mitigating the impact of an emergency response on an affected population. Performance management of individuals and teams is an important issue to improve and monitor the quality of emergency response. Performance management through such tools as after action reviews and outbreak response debriefs have been shown to improve team performance by 20–25% [27]. Implementation of these tools, however, are often conducted after the response.

Research has identified that emergency response setting has multiple challenges that directly affect teams and their performance. These challenges include the identification, availability, and selection of suitable individuals, high staff rotation and short contracts, minimal understanding of roles, and limited collective competence amongst teams [4, 12, 28]. Considering Tuckman’s team lifecycle within the context of these challenges, monitoring of human resources during emergency response is needed [6]. Teams need support to form and better collaborate as a team during a high stress, fast moving event. Performance management of individuals and the team as a whole may support this progress.

Given the unusual environment and known team dynamic challenges within emergency response settings, teams would benefit from the development and implementation of a real time (or close to it), customised team performance tool.

### Leadership and team effectiveness

Managing emergency response settings requires leadership and collaboration [13]. The impact of team dynamics, team confidence, and team management during emergency response influence the team effectiveness, and in turn impact the effectiveness of response [12, 13]. The stress of a crisis, however, often makes teamwork and collaboration difficult to sustain [29, 30].

Tuckman’s team lifecycle is important to consider in regards to leadership also [6]. Emergency response leaders need skills in managing a fluctuating team through a high stress, fast moving event. Due to the identified team challenges, response leaders need to acknowledge that their team are unlikely to progress to, or maintain a high level of performance. For this reason, response leaders need strategies to better lead a team through the early team phases identified in Tuckman’s team phases.

Strong and clear leadership is critical for team functionality. However, our findings indicate leadership is often lacking in the emergency response setting for public health response. Our study identified consistent differences in the perception of leadership by gender. We also identified that national responders consistently rated their team performance and leadership as higher than the international responders in the survey, across all categories. Leadership and team interaction are often culturally and contextually bound [31]. Satisfaction in leadership is relative to expectations of leadership, this is an important finding to test in future studies. Different styles of leadership have been shown to have a positive impact on individuals as well as team satisfaction, this would be an important element to review in future studies also [32, 33].

An important aspect of teamwork identified in this study was enjoyment. Emergency response is often conducted under difficult conditions, with enormous time pressures, and high risk for the impacted community as well as the response team. The ability to laugh and keep morale high was reported by participants as needed and appreciated to keep team morale high.

Given the complexity of emergency response, any future team performance tool will need to look beyond leadership, teamwork, and task management. The dynamic nature of team composition adds a level of complexity which those three themes do not capture. Any future performance tool would need to take these issues into consideration. Our research identified that an effective team is collectively competent, with a mix of experience, skills, and perceptions. Clear roles and communication are essential, as is a sense of belonging and purpose [34, 35]. A team needs a mix of people with experience in emergency response, to enable mentorship, learning, and direction [36]. An emergency response team also require team resilience and ‘productive divergence’, with differences of opinion to help energise a team [7]. Critically, team effectiveness during emergencies relies on strong and clear leadership. Our findings on leadership corroborate previous research that indicates team effectiveness and innovation improve when leadership is agile to team needs and clear with team roles [4, 7, 12, 37]. Table 5 outlines our findings in this paper against Tuckman’s team lifecycle [6].

**Table 5:** Findings applied to Tuckman’s team lifecycle

Forming	Storming	Norming	Performing	Adjourning
- Facilitation of opportunities for team members to build relationships - Establishment of clear expectations - Strong leadership with clear direction - Adjusting to continual changes in team composition	- Early identification and recognition of issues - Facilitation of resolution - Building trust - Encouraging feedback - Supportive environment with opportunity for debriefing - Identification of knowledge/skill gaps - Adjustment of roles / responsibilities based on skill sets	- Recognition of individual skills / knowledge - Mentoring - Strengthening skills / knowledge with learning opportunities and feedback - Building a positive team working culture - Recognition of what each person brings to the team	- Clear roles and responsibilities - Celebrating success - Providing opportunity for collective problem solving and decision making - Flexibility to adjust to rapid changes	- Provide opportunity for reflection on team function and performance - Celebrate positive outcomes - Identify and share lessons learnt - Identify teams or individuals that work well together for future response collaboration

### Limitations

This survey was conducted before the COVID-19 pandemic, however the findings are equally as relevant now. A study limitation was obtaining an accurate denominator for the target population as the number of responders and scale of emergencies change from year-to-year with workforce shifting according to need and organisational funding.

There were varying timeframes between emergency response and survey completion amongst survey participants. Participants may therefore have different levels of recall, and people with extremely negative or positive response experiences may remember differently. Participants may have had differing levels of expectation of their team and leadership, this was unable to be tested in this study.

There was a potential for selection bias as it was self-administered online. We attempted to lessen the impact of this bias by using multiple pathways to recruit participants and collaborated closely with emergency response partners. As the survey was available in French and English, this aimed to increase representation from a variety of people and contexts, however this may have missed valuable information from other language groups.

## Conclusion

Our study provides further evidence that collective competence is needed and leadership skills are vital for teams to effectively respond to emergencies. We recommend further research to understand the gender differences identified in this study. We also recommend development of a team performance tool for simple continuous mid-response assessment, to ensure issues can be addressed proactively and for leaders to better understand and manage the team dynamics especially within international teams.

## Data Availability

All data relevant to this manuscript are included within the manuscript tables, figurers and/or reference links.

## Abbreviations

FETP: Field Epidemiology Training Program
SD: Standard deviation
TEAM: Team Emergency Assessment Measure
TEPHIConnect: Training Programs in Epidemiology and Public Health Interventions Network alumni
WHO: World Health Organization
